# Intra-patient dose escalation in Ewing’s sarcoma treated with vincristine, doxorubicin, cyclophosphamide alternating with ifosfamide and etoposide: a retrospective review

**DOI:** 10.1186/2045-3329-3-15

**Published:** 2013-12-10

**Authors:** Jeremy Lewin, Samantha Wieringa, Marnie Collins, Jayesh Desai, Lisa Orme, Senthil Lingaratnam, David M Thomas

**Affiliations:** 1 Sarcoma Service, Peter MacCallum Cancer Centre, Locked Bag 1, A’Beckett Street, Melbourne, VIC 8006, Australia; 2 Pharmacy Department, Peter MacCallum Cancer Centre, Melbourne, Australia

**Keywords:** Ewing’s sarcoma, Dose escalation, Toxicity, Neutropenia, Chemotherapy

## Abstract

**Background:**

Data suggests that males experience less toxicity and poorer survival than females treated for Ewing’s sarcoma. We instituted an intra-patient dose escalation (DE) policy with Vincristine/Doxorubicin/Cyclophosphamide (VDC) alternating with Ifosfamide/Etoposide (IE) based on hematological nadirs and report its feasibility and safety.

**Methods:**

A retrospective review of adherence to DE guidelines and toxicities was conducted for patients who received DE with VDC/IE over 3 years at a single cancer center. Absolute neutrophil counts (ANC) was collected on days 8, 12 and 15 for cycles 1–6. DE of 10%/cycle was applied if ANC > 1.5×10^9^/L and platelet > 100×10^9^/L on all blood results. The primary endpoint was the proportion of patients who received appropriate DE. The secondary endpoint was to assess morbidity, changes in hematologic nadirs between gender and age and a comparison with a prior cohort of ESFT patients who did not receive DE. Gender comparisons were assessed via independent 2-sample t-tests assuming unequal variances. Within cycle changes in hematologic nadirs were assessed using repeated measures ANOVA. Relapse free survival and overall survival (OS) curves were estimated using the Kaplan-Meier method.

**Results:**

23 patients were identified (mean age: 27; range 17–54). 91 decisions for DE were made (1 decision excluded because of progressive disease) with 90% concordance with guidelines. No adverse outcomes occurred as a result of the inappropriate escalation. Grade 3/4 febrile neutropenia (FN) during VDC and IE was 26.1% (6/23 patients) and 17.4% respectively with no difference for those who were DE. Males were less neutropenic after C1 and C3 of VDC compared to females (P-value C1 = 0.003; C3 = 0.005). VDC was associated with greater neutropenia on day 8 whereas IE had greater neutropenia on day 12 (P-value <0.001). During VDC, a non statistical difference in neutropenia was seen for individuals aged 15–25 (n = 13) compared with older individuals (P-value = 0.09). OS comparison for those with localized disease with a prior cohort who were not DE showed similar outcomes (P-value = 0.37).

**Conclusions:**

DE is deliverable without increased adverse outcomes. Males have less myelosuppression during VDC, and should be especially considered for DE.

## Background

Ewing sarcoma family tumors (ESFT) are malignant tumors of bone and soft tissue. Patients presenting with localized disease are usually treated with multimodality approach, given high relapse rates (80-90%) without chemotherapy [1,2]. Modern treatment plans include neo-adjuvant and adjuvant multi-agent chemotherapy, to target the high risk of subclinical metastatic disease at the time of diagnosis. In studies predominantly involving pediatric populations, this approach leads to 5 year survival rates around 70% [3-5]. Even in patients presenting with metastatic disease, long-term survival may be seen in 20% of patients [2]. The traditional chemotherapy used in both localized and metastatic disease is Vincristine, Doxorubicin, Cyclophosphamide alternating with Ifosfamide and Etoposide (VDC/IE) [2,6], with suggestions that the dose of doxorubicin may be related to outcome [7].

Despite a steady improvement in cancer care generally, there has been a discouraging lack of improvement in survival rates in adolescent and young adults (AYA) [8]. For patients diagnosed with ESFT between the ages 15–30, survival is approximately half that of children with males having increased mortality compared to females in the AYA subgroup [9-11]. There has been much speculation as to the reasons for these observations. The suggestion that relative underdosing of AYA compared to children may play a part is supported by data showing that adults with pediatric-type sarcomas experience less treatment-related toxicity compared with their younger counterparts [10,12-14]. A meta-analysis in osteosarcoma showed that children had higher rates of neutropenia compared with adolescent or adult patients, and that dose-related toxicities including thrombocytopenia and mucositis were strongly associated with improved overall survival [9]. In addition to age, gender-related differences also exist in osteosarcoma, with males experiencing less myelosuppression and inferior survival [7,9]. A retrospective study of 14,824 patients showed that male AYA patients with osteosarcoma, Ewing sarcoma and Hodgkin lymphoma experienced less toxicity, lower response rates and excess mortality relative to children and female counterparts [12]. Similar findings correlating lack of chemotherapy-induced toxicity and inferior outcome [15-17], have also been reported in testis, breast and ovarian cancers [18].

These data suggest that age and gender-related differences in drug handling may account for some of the survival and toxicity differences in the AYA population. If so, dose intensification may improve outcomes in chemosensitive cancers. Accurate dosing of chemotherapy is difficult using traditional algorithms based on body surface area [19], with evidence for systematic underdosing [20,21]. While one study of dose escalation of alkylating agents did not improve outcomes in Ewing sarcoma [22], increasing dose density from a 21 day cycle to a 14 day cycle with growth factor support [23] may be associated with improved survival without increased toxicity [24]. These approaches have relied on dose intensification across all patients treated on these protocols, rather than intra-patient dose modification to achieve a pharmacodynamic effect. Current studies of anthracycline pharmacokinetics and pharmacodynamics (ACTRN12609000956202) will aid in justifying and developing strategies for “individualizing” chemotherapy, enabling the use of readily available surrogate markers such as the degree of myelosuppression, or measures of the antiproliferative activity of drugs, to guide dose-adjustments [25,26].

In the absence of more accurate algorithms for chemotherapy dosing, one practical solution for personalized, intra-patient dosing is “toxicity-adjusted” dosing [20]. In 2009, we implemented a policy at the Peter MacCallum Cancer Centre to individualized dose escalation of chemotherapy in Ewing sarcoma patients, using nadir bloods counts as the primary measure of a dose-related pharmacodynamic effect. We now report on our dose escalation (DE) policy over a 3 year period to assess overall deliverability, safety and treatment related toxicity.

## Methods

The primary objective was to assess the proportion of patients that received DE and the proportion of DE decisions that were appropriate according to the protocol. The secondary objective was to assess rates of serious complications for the entire group, investigate gender and age related differences in neutrophil and platelet nadirs, assess subsequent neutrophil and platelet nadirs for those who underwent dose escalation, and calculate relapse free survival (RFS) and overall survival (OS). In order to assess RFS and OS, a comparison was performed with a prior cohort of ESFT with localized disease treated with the same regimen of VDC/IE, but prior to the initiation of our dose escalation policy. This study was approved by the Institutional Review Board of Peter MacCallum Cancer Centre for retrospective data collection.

Chemotherapy was delivered for ESFT every 3 weeks with vincristine (2 mg), doxorubicin (75 mg/m^2^), cyclophosphamide (1200 mg/m^2^) D1 alternating with ifosfamide (1800 mg/m^2^) and etoposide (100 mg/m^2^) D1-5 [2]. Blood counts were taken on Day 8, 12 and 15 for the first 6 cycles of chemotherapy and dose escalation (DE) was implemented if the neutrophil and platelet count did not fall below 1.5×10^9^/L or 100×10^9^/L, respectively, in the absence of non-hematologic toxicities of concern. Although these hematological cut-offs have not been validated in prospective trials, these values have been suggested in the literature in recommendations for “toxicity adjusted dosing” [15,18]. Dose escalation involved increasing the BSA calculated dose of all chemotherapy agents, except vincristine, by 10% for all equivalent subsequent cycles. Given the alternating regimen, nadir counts for cycle 1 and 2 led to DE decision for cycle 3 and 4, whereas nadir counts for cycle 3 and 4 led to a DE decision in cycle 5 and 6. De-escalation occurred on the basis of both hematological and non-hematological toxicities at the discretion of the treating physician.

### Inclusion

All patients with ESFT (including peripheral primitive neuroectodermal tumor, Askin’s tumors, Ewing’s sarcoma, desmoplastic small round cell tumor) who underwent cytotoxic chemotherapy at Peter MacCallum with VDC/IE between March 2009 and August 2012 were included.

### Statistical analysis

Analysis of primary endpoints and secondary endpoints was performed on an intention to treat basis. To address the primary objective, the proportion of patients who received DE was calculated for each of cycles 3 – 6 separately as well as for any VDC cycle (3 and/or 5) and any IE cycle (4 and/or 6). To evaluate adherence, the proportion of treatment decisions that were appropriate according to the DE protocol was calculated.

To address the secondary objectives, the proportion of patients reporting grade 3/4 febrile neutropenia in each of the VDC and IE cycles was calculated. Comparisons between males and females in terms of neutrophil and platelet nadirs in cycles 1 – 4 were investigated using independent 2-sample t-tests assuming unequal variances. Within cycle changes in neutrophil and platelet counts as well as between cycle changes in neutrophil and platelet nadirs were assessed using repeated measures ANOVA. Overall survival was measured as the time from first chemotherapy until death and relapse-free survival was measured from time of first chemotherapy until date of relapse. Patients were censored at the last date they were known to be alive and relapse-free respectively. OS and RFS curves were estimated using the Kaplan-Meier product-limit method. OS and RFS comparison to the prior historical cohort was assessed using the logrank test. A *P*-value of <0.05 was considered statistically significant. Statistical analysis was performed using Excel, GraphPad Prism 6 and R version 2.15.2 software.

## Results

Between 2009 and 2012, 23 patients were treated on the DE protocol with a median follow up of 2.3 (0.7 – 3.4) years. Baseline demographics are shown in Table 1. In total, 91 events for consideration of dose escalation occurred (1 decisions was excluded because of progressive disease). This is presented in Table 2. There were 3 patients during VDC that were escalated twice. Although not statistically significant, more males had DE than females during VDC (7/13 vs 3/10), but not during IE (7/13 vs 5/10).

**Table 1:** Baseline demographics

**Baseline demographics**	
Number	23
Female	10 (43%)
Male	13 (57%)
Median age (range)	23 (17 – 54)
Median age female	21 (17 – 54)
Median age male	26 (18 – 40)
Diagnosis	
EWINGS	17 (74%)
PNET	5 (22%)
DSRCT	1 (4%)
Disease site	
Metastatic	6 (26%)
Localized	
Extremity	9 (39%)
Pelvic	2 (9%)
Axial trunk	2 (9%)
Chest wall	3 (13%)
Other	1 (4%)
BSA	
Female	1.79 (SD ± 0.22)
Male	1.94 (SD ± 0.15)

**Table 2:** Number of patients who received dose escalation during VDC/IE

	**Escalated**	**Dose unchanged**	**Dose reduced**	**Number**
VDC				
C1				23
C3	10	10	3	23
C5	3	19	1	23
Any cycle	10	10	3	23
IE				
C2				23
C4	10	11	2	23
C6	3	19	0	22
Any cycle	12	9	2	23

Protocol adherence was recorded at 90%. Nine events of non-adherence to the protocol were recorded (7 events of inappropriate escalation (7.7%) and 2 events of missed escalation (2.2%); details of inappropriate escalation are shown in Additional file 1: Table S1). No serious adverse outcomes occurred as a result of the inappropriate escalation. The rate of Grade 3/4 febrile neutropenia (FN) during VDC was 26.1% (6/23 patients) with median length of stay (LOS) of 4 days (range 1–20) with no ICU admissions. The rate of Grade 3/4 FN for IE was 17.4% (4/23 patients) with a median LOS of 4 days (3–7) and 1 ICU admission. The ICU stay occurred in a patient who was appropriately not dose-escalated. There were no deaths related to chemotherapy and only 1 patient received a platelet transfusion (during IE). There were no additional documented grade 3/4 toxicities documented in those who underwent dose escalation.

Neutrophil nadirs by gender for cycle 1 – 4 are shown in Table 3. Males were statistically significantly less neutropenic after C1 and C3 of VDC compared to females (*P*-value C1 = 0.003; C3 = 0.005). VDC was associated with greater neutropenia on day 8 (ANC: D8: 0.98 ± 0.97 × 10^9^/L, *P*-value <0.001) whereas IE had greater neutropenia on day 12 (ANC: D12: 3.74 ± 3.04 × 10^9^/L, *P*-value <0.001). Although not statistically significant, there was also a trend for less thrombocytopenia during VDC in males (data not shown, *P*-value = 0.11). After cycle 1 of VDC, individuals aged 15–25 (n = 13) had lower neutrophil nadirs compared with older individuals (mean difference = 0.62 × 10^9^/L, *P*-value = 0.15) although this was not statistically significant. Differences in cytopenia in IE dosing according to gender were only seen in cycle 4 (mean difference = 2.31 × 10^9^/L, *P*-value = 0.05) (Table 3).

**Table 3:** Gender related difference in nadir blood counts after C1 and C3

	**Gender**						**Mean difference**	**95% CI**	***P*-value**
	**Male**			**Female**					
	***N***	**Mean**	**SD**	***N***	**Mean**	**SD**			
Neutrophil nadir									
VDC									
Cycle 1	13	1.56	1.35	9	0.16	0.19	1.40	(0.57, 2.21)	0.003
Cycle 3	12	1.59	1.08	9	0.42	0.56	1.17	(0.40, 1.93)	0.005
IE									
Cycle 2	13	3.77	2.70	10	4.40	4.59	−0.63	(−4.14, 2.88)	0.71
Cycle 4	12	3.17	3.54	9	0.86	1.18	2.31	(−0.04, 4.66)	0.05
Dose intensity (%)									
VDC	13	107	9	10	98	16	9	(−3, 22)	0.12
IE	13	105	7	10	102	11	3	(−5, 11)	0.49

*P*–values correspond to comparisons between genders. Dose intensity of VDC and IE is compared to baseline.

Although the numbers were too small to achieve statistical significance, dose escalation, whether appropriate or not, was associated with a reduction in neutrophil count in subsequent cycles. For those who received dose escalation in cycle 1 of VDC, the mean nadir ANC decreased from 1.59 ± 1.22 × 10^9^/L to 1.29 ± 1.07 × 10^9^/L in cycle 3, and 1.04 ± 0.9 × 10^9^/L in cycle 5 (*P*-value = 0.29). For IE cycles, those who received dose escalation after cycle 2 showed a decrease from 4.82 ± 1.88 × 10^9^/L to 2.10 ± 1.8 × 10^9^/L in cycle 4. Using current dosing algorithms, dose escalation resulted in a trend to males receiving a higher relative dose intensity of VDC and IE compared to females (VDC: 107% vs 98%, *P*-value = 0.12; IE: 105% vs 102%, *P*-value = 0.49).

**Figure 1:**
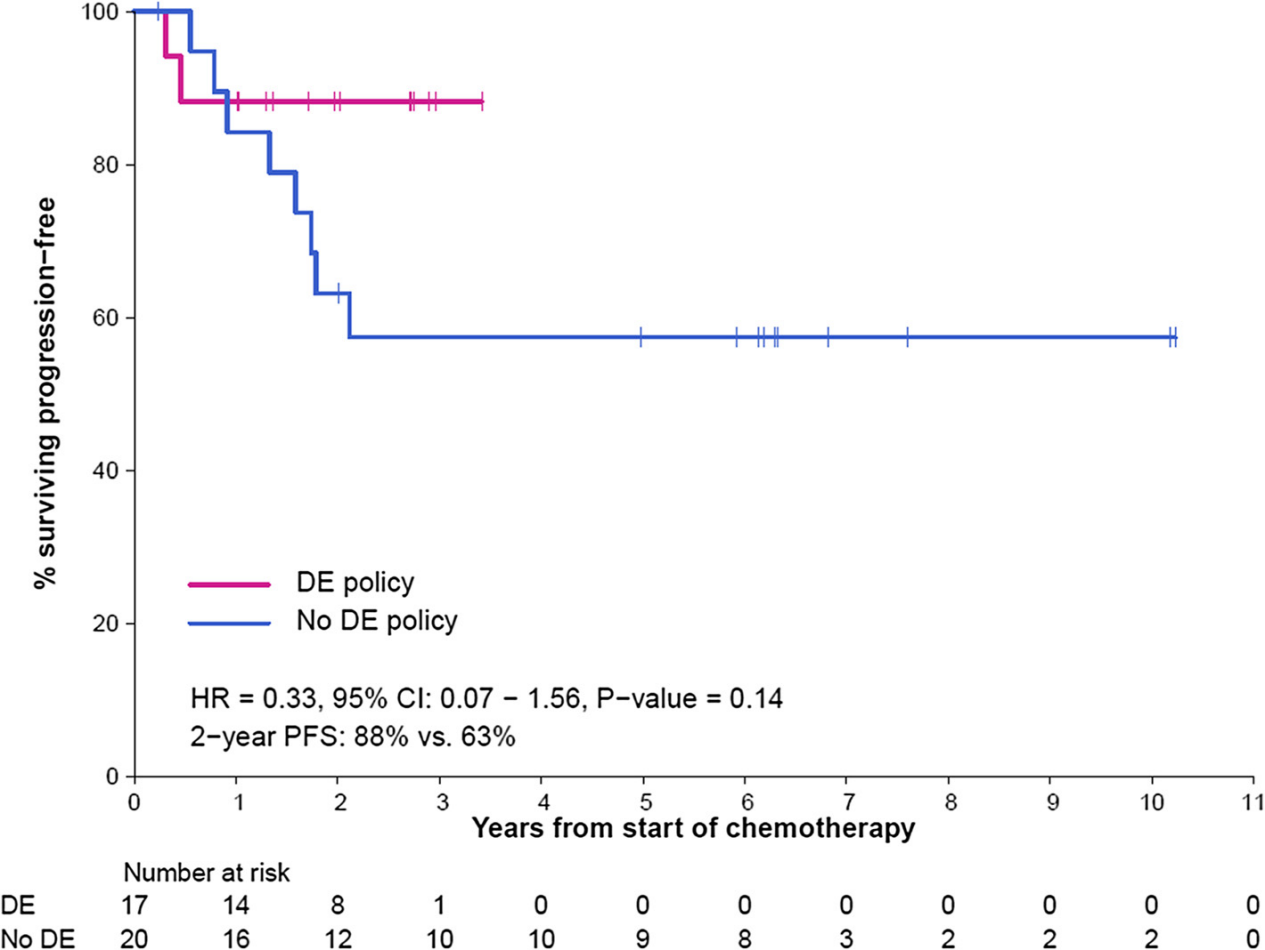
**Relapsed-free survival for localized ESFT compared between those who underwent a DE policy (2009 – 2012) compared with a prior cohort who did not have a DE policy (1995 – 2004) showing a HR = 0.33 (95% CI: (0.07 – 1.53) *P-*** **value 0.14).**

**Figure 2:**
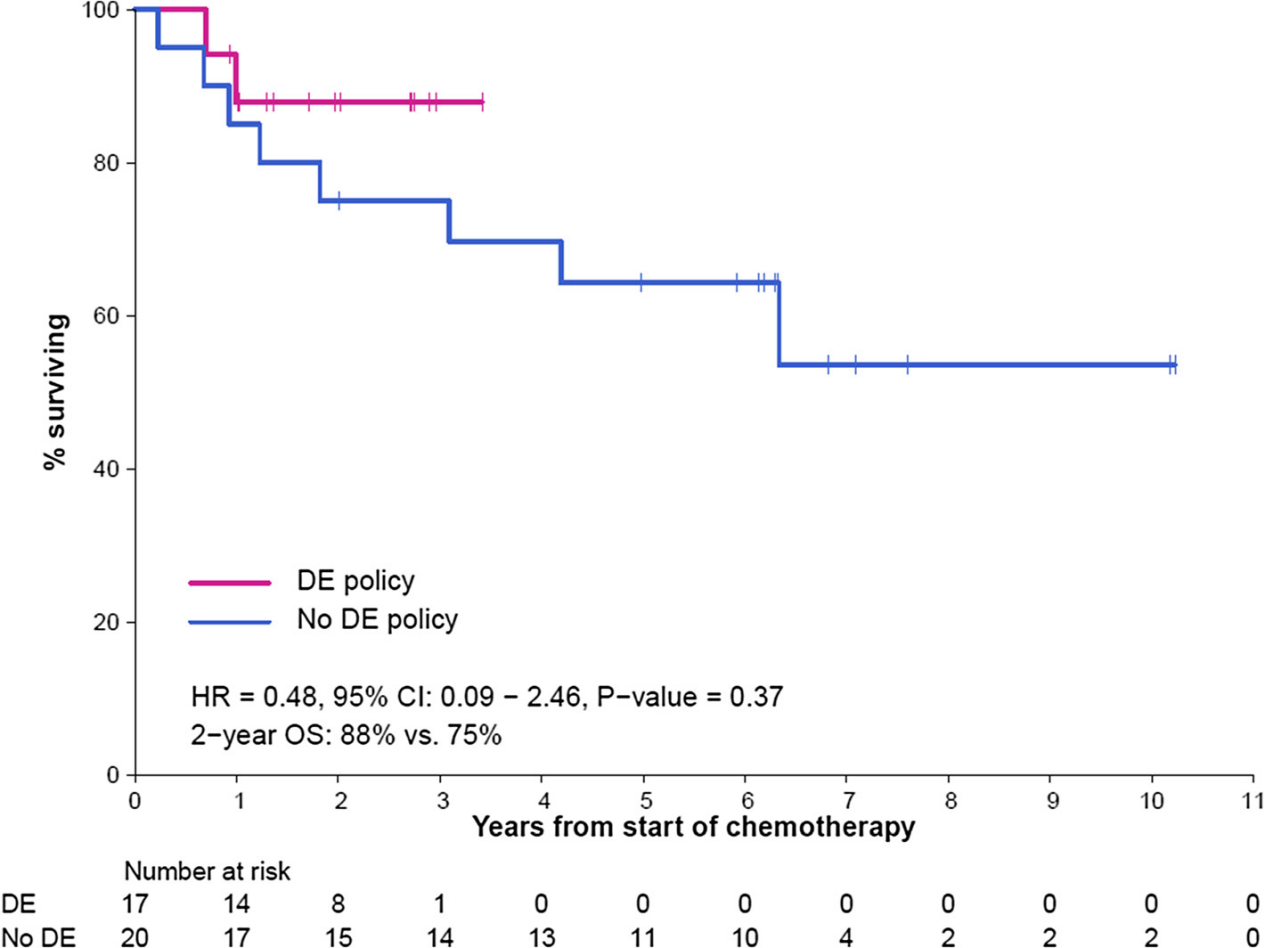
**Overall survival for localized ESFT compared between those who underwent a DE policy (2009 – 2012) compared with a prior cohort who did not have a DE policy (1995 – 2004) showing a HR = 0.48 (95% CI: (0.09 – 2.46) *P-*** **value 0.37).**

To compare overall cancer-related outcomes between the traditional BSA associated chemotherapy dosing and the toxicity-adapted dose escalation protocol, we compared the RFS and OS between treated patients with localized disease before (1995–2004) and during (2009–2013) the introduction of the DE policy. The baseline characteristics of this historical comparator group were similar to our cohort (An additional file shows this in more detail [see Additional file 2: Table S2]) and patients with DSRCT and metastatic disease were excluded from this analysis. The 2 year RFS in the dose escalation cohort was 88%, compared to 63% in the historical comparator cohort (HR = 0.33, 95% CI: (0.07-1.56); *P*-value = 0.14) and 2 year overall survival was 88% vs 75% in the historical comparator cohort (HR = 0.48, 95% CI: (0.09-2.46) *P*-value = 0.37 (Figures 1 and 2). When making comparisons in our cohort group only, there was no statistically significant difference in RFS (*P*-value = 0.29) and OS (*P*-value = 0.31) when separated by those who have had DE (for either VDC or IE) compared to those who did not in patients with localized disease.

## Discussion

The key finding in this study is that individualized dose escalation of chemotherapy in ESFT is feasible in a single sarcoma unit. Less neutropenia was observed in males, and perhaps in older patients, during the anthracycline cycles, suggesting that BSA-based dosing alone may not be adequate in males. The protocol compliance rate was 90%. Given the experimental nature of a toxicity-adjusted protocol, we continually reviewed the safety and compliance after implementation of the protocol. Importantly, no short or long term adverse outcomes were seen in this study as a result of intrapatient dose escalation. The RFS and OS comparison to the prior ESFT cohort suggests that dose escalation is not associated with worse overall outcomes for patients with localized primary ESFT. Clearly, safe dose escalation requires a formal program with strictly defined criteria for escalation, close monitoring of nadir blood counts, and patient selection limited to those without other dose-related toxicities or co-morbid illnesses. Given this study showed gender and age related differences in cytopenia, it is arguable that dose escalation should be especially considered in older males during the anthracycline component of treatment (Cycle 3 or 5 based on C1 nadir). Additionally, variation was seen in the timing of neutropenia nadirs, which may have ramifications for the timing of pegylated filgrastim delivery.

It is important to put these findings into context given evidence for gender- and age-related difference in toxicity and outcome in chemo-sensitive cancers such as ESFT [12]. Although the peak incidence of ESFT is during the AYA years, these patients have a worse survival compared to a pediatric population [27]. The reasons behind this comprise a combination of biological differences [27,28], delayed diagnosis, poorer treatment compliance [29,30], psychosocial overlay and limited involvement in clinical trials [31,32]. With regards to biological differences, in the EURO-Ewing 99 protocol [10], increasing age and male gender was associated with less toxicity using VIDE chemotherapy, a protocol similar to VDC/IE. Additionally, gender related differences have previously been described in hodgkins lymphoma [33], osteosarcoma [9,34,35] and ewings sarcoma [12,36,37]. A meta-analysis in osteosarcoma confirmed differences in outcome related to gender and age and showed improved outcomes in females, and those with Grade 3/4 mucositis [9]. Interestingly, it also confirmed the results of our study showing increased myelosuppression in the younger age group although given our small numbers, this was not statistically significant. Collectively, this data suggests a better understanding of cytotoxic pharmacology is needed in the AYA population [28,38], and may be used to improve cancer outcomes.

Adolescent and young adult patients undergo gender-specific pubertal changes in body composition, size and hormonal status, which may alter the pharmacokinetic/pharmacodynamic properties of chemotherapy [38]. Young women have significant increase in fat mass during adolescent years, with subsequent higher body fat than men [39]. Higher fat distribution has effects on drug clearance and volume of distribution [28,40] which may cause variations in the dose of chemotherapy delivered. That being said, a large pediatric study in ALL showed no differences in toxicity, survival or PK data in the obese patient [41]. It is difficult to isolate drug-specific effects in complex, multi-drug chemotherapeutic regimens, and it is important to recognize that different drugs may demonstrate different pharmacologic profiles in the AYA population. Nearly all the benefit of methotrexate’s use in osteosarcoma has been demonstrated in patients under the age of 40 [42] and doxorubicin, a key component of regimens used to treat ESFT, osteosarcoma and hodgkins disease, demonstrates significant pharmacokinetic variation according to gender and body habitus [43,44]. It is possible that the inferior outcomes of EFST in older males may be attributed to relative doxorubicin under-dosing of these individuals.

Given the inherent inaccuracies in current dosing algorithms, and the importance of chemotherapy to cure for chemo-sensitive cancers, intra-patient dose modification based on markers of toxicity provides a practical strategy for individually tailored dosing [25,26]. In this context, this limited study suggests gender- and potentially age-related differences in hematological nadirs, and that intra-patient dose escalation appears safe and feasible. It is important to note that this retrospective study only comprises 23 patients, with relatively short follow up. In particular, the non-significant trend to improved RFS and OS in the dose escalation group will require larger numbers of patients and longer follow up. Additionally, we did not assess long-term morbidity, including the potential for cumulative anthracycline cardiotoxicity in a young population.

## Conclusion

Individualized, toxicity-adapted dosing of chemotherapy, added to an initial calculation of dosing based on BSA-alone, aims to correct the gender and age related disparity in outcomes, without relying on new chemotherapeutic agents. Pharmacological data in this age group is lacking [28] and adequate dose–response is critical for chemo-sensitive diseases such as ESFT, germ cell tumors and Hodgkin’s in maximizing outcome where a dose–response relationship exists. The key finding in this study is that individualized dose escalation of chemotherapy in ESFT is feasible in a single sarcoma unit without increased adverse outcomes. Additionally we have showed that less neutropenia was observed in males, and perhaps in older patients, during the anthracycline cycles and should be especially considered for DE. Given the rarity of ESFT presents significant statistical challenges in stratifying gender and age differences, pharmacological endpoints should be designed into future prospective chemotherapy trials.

## Abbreviations

ANC: Absolute neutrophil count; AYA: Adolescent and young adult; DE: Dose escalation; ESFT: Ewing’s sarcoma family tumors; FN: Febrile neutropenia; ICU: Intensive care unit; IE: Ifosfamide, etoposide; VDC: Vincristine, doxorubicin, cyclophosphamide; LOS: Length of stay; OS: Overall survival; RFS: Relapse free survival.

## Competing interests

The authors declare that they have no competing interests.

## Authors’ contributions

JL and DT conceived and designed the study. SW and JL undertook the data retrieval. JL, DT and MC analyzed the data. All authors participated in writing of the manuscript. All authors read and approved the final manuscript.

## Supplementary Material

Additional file 1: Table S1 Description of patients who inappropriately received dose escalation. Click here for file

Additional file 2: Table S2 Comparison of baseline demographics with for patients with localized disease who underwent a DE policy (2009 – 2012) compared with a prior cohort who did not have a DE policy (1995 – 2004). Patients in both cohorts were treated with alternating VDC/IE. Click here for file
