# Supplementary material for: Intra-patient dose escalation in Ewing’s sarcoma treated with vincristine, doxorubicin, cyclophosphamide alternating with ifosfamide and etoposide: a retrospective review
Source: Clin Sarcoma Res. 2013 Dec 10;3:15. doi: 10.1186/2045-3329-3-15 (PMC3866566; doi:10.1186/2045-3329-3-15)
Supplement: Additional file 1: Table S1 — Description of patients who inappropriately received dose escalation. [file 2045-3329-3-15-S1.doc]

| **Patient Details** | **Neutrophil Nadir**  **(× 10^9^/L)** | **Platelet Nadir**  **(× 10^9^/L)** | **Dose escalation (%)** | **Outcome (eg FN, ICU stay, plt transfusion)** |
| --- | --- | --- | --- | --- |
| **VDC component** | | | | |
| 1. **23 yo female with localized disease of pelvis sub-optimally debulked with residual disease** | 0.6 | 217 | 10% | Calculated dose intensification given disease. No adverse outcome with subsequent neutrophil nadir in C3 of 0.3. Patient had initial response but progressive disease on VDC/IE after 5 cycles |
| 1. **21 yo female receiving neo-adjuvant therapy for Ewing’s of R thigh** | 0.0 | 148 | 10% | Unclear escalation as no documentation but neutrophil nadir was on external bloods. No adverse outcome with subsequent neutrophil nadir in C3 of 0.2. Patient had initial partial response after 4C of neo-adjuvant chemotherapy. Surgery deferred due to proximity to neurovascular bundle. After 8 cycles had progressive disease. |
| 1. **18 yo male receiving neo-adjuvant therapy for Ewing’s of L tibia** | 0.52 | 110 | 10% | Unclear escalation as no documentation. No adverse outcome with subsequent neutrophil nadir of 3.21 in C3 and 0.37 in C5 |
| 1. **24 yo male receiving treatment for metastatic DSRCT** | 0.9 | 138 | 10% | Unclear escalation as no documentation. No adverse outcome with subsequent neutrophil nadir of 1.41 on C3 and 1.38 in C5 |
| **IE component** | | | | |
| 1. **40 yo male receiving treatment for metastatic Ewing’s** | 6.4 | 70 | 10% | Unclear escalation as no documentation. No adverse outcome with subsequent neutrophil nadir of 0.5 in C4 and 0.0 in C6. Platelet nadir was 42 in C4 and 30 in C6 |
| 1. **18 yo male receiving neo-adjuvant therapy for Ewing’s of L tibia (same as pt 3)** | 3.38 | 79 | 5% | Calculated escalation for dose intensity. No adverse outcome with subsequent neutrophil nadir of 2.55 in C4 and 4.8 in C6. Platelet nadir was 74 in C4 and 63 in C6 |
| 1. **17 yo female receiving neo-adjuvant therapy for Ewing’s of L thigh** | 5.2 | 92 | 10% | No adverse outcome with subsequent neutrophil nadir of 0.4 in C4 and 2.0 in C6. Platelet nadir was 137 in C4 and 128 in C6 |

**Additional file 1: Table S1:** Description of patients who inappropriately received dose escalation**.**
