# Supplementary material for: Intra-patient dose escalation in Ewing’s sarcoma treated with vincristine, doxorubicin, cyclophosphamide alternating with ifosfamide and etoposide: a retrospective review
Source: Clin Sarcoma Res. 2013 Dec 10;3:15. doi: 10.1186/2045-3329-3-15 (PMC3866566; doi:10.1186/2045-3329-3-15)
Supplement: Additional file 2: Table S2 — Comparison of baseline demographics with for patients with localized disease who underwent a DE policy (2009 – 2012) compared with a prior cohort who did not have a DE policy (1995 – 2004). Patients in both cohorts were treated with alternating VDC/IE. [file 2045-3329-3-15-S2.doc]

| **Baseline Demographics for localized disease** |  |  |
| --- | --- | --- |
| **Cohort** | **2009-2012** | **1995-2004** |
| **Number** | **17** | **20** |
| **Female** | **8 (47%)** | **9 (45%)** |
| **Male** | **9 (53%)** | **11 (55%)** |
| **Median Age (Range)** | **23 (17-54)** | **24 (17-40)** |
| **Median Age Female** | **21 (17-54)** | **19 (17-40)** |
| **Median Age Male** | **26 (18-40)** | **26 (17-35)** |
| **Diagnosis** |  |  |
| **EWINGS** | **13 (76%)** | **20 (100%)** |
| **PNET** | **4 (24%)** |  |
| **Disease site** |  |  |
| **localised** |  |  |
| **Extremity** | **9 (53%)** | **11 (55%)** |
| **Pelvic** | **2 (12%)** | **4 (20%)** |
| **Axial trunk** | **2 (12%)** | **3 (15%)** |
| **Chest wall** | **3 (17%)** | **1 (5%)** |
| **Other** | **1 (6%)** | **1 (5%)** |

**Addditional file 2: Table S2:** Comparison of baseline demographics with for patients with localized disease who underwent a DE policy (2009 – 2012) compared with a prior cohort who did not have a DE policy (1995 – 2004). Patients in both cohorts were treated with alternating VDC/IE.
